# Warning line for preventing bone cement leakage in surgery involving percutaneous kyphoplasty for osteoporotic vertebral compression fractures

**DOI:** 10.3389/fsurg.2025.1530495

**Published:** 2025-04-11

**Authors:** Dongyue Li, Luming Tao, Qingjun Su, Xinuo Zhang, Xingrui Wu

**Affiliations:** Orthopaedic Department, Chaoyang Hospital Affiliated with Capital Medical University, Beijing, China

**Keywords:** percutaneous kyphoplasty (PKP), osteoporotic vertebral compression fractures (OVCFs), bone cement, leakage, warning line

## Abstract

**Background:**

Percutaneous kyphoplasty (PKP) has achieved good clinical efficacy in the treatment of Osteoporotic vertebral compression fractures (OVCFs). However, how to reduce the bone cement leakage rate and improve safety during PKP surgery remains an urgent issue to be addressed in clinical practice. Therefore, the aim of this study was to identify a line, called the “warning line”, to determine whether there is leakage of bone cement during PKP surgery.

**Methods:**

From February 2018 to September 2022, 88 patients and 106 vertebral bodies with OVCFs treated with PKP by a single surgeon at our center were included in the study. Clinical general data were recorded. Vertebral bodies with bone cement reaching the apex of the posterior margin depression without leakage were designated Group A, whereas those with leakage were designated Group B. The posterior vertebral wall was divided into three equal parts in the postoperative three-dimensional CT scans, and the leakage rates at different positions of the posterior vertebral wall were analysed. In Group A without leakage, line b, called the warning line, was marked as the apex of cement diffusion.

**Results:**

All 88 patients successfully underwent surgery, with a significant decrease in the postoperative VAS score. No neurological complications occurred. Bone cement leakage rate was 58.5%. There were 44 vertebral bodies in Group A and 62 in Group B. No significant differences were found between the two groups in terms of age, bone density, balloon pressure, contrast dose, or cement volume (*P* > 0.05). The bone cement leakage rates in the upper third, middle third, and lower third of the posterior vertebral wall were 25%, 61.1%, and 66.7%, respectively, with statistically significant differences (*P* < 0.05). In Group A without leakage, the warning line was approximately 6.8% of the sagittal diameter from the posterior vertebral margin.

**Conclusions:**

PKP is a relatively safe treatment for OVCFs. Most bone cement leakage occurs in the middle and lower thirds of the posterior vertebral wall. When the apex of cement diffusion remains anterior to the warning line in PKP surgery, the posterior vertebral wall cement leakage rate is low.

## Introduction

With the increasing aging of society, Osteoporotic vertebral compression fractures (OVCFs) have gradually become a serious problem affecting the quality of life of elderly individuals (1). In recent years, numerous clinical studies have confirmed the good clinical efficacy of percutaneous kyphoplasty (PKP) in treating OVCFs, and PKP has been widely used for this purpose. However, bone cement leakage remains the main complication of PKP surgery (2–5). Although most cases of bone cement leakage are asymptomatic, some can lead to catastrophic outcomes. For example, intravertebral canal leakage of bone cement could cause varying degrees of damage to the spinal cord and nerves due to direct compression or exothermic reactions during cement polymerization, resulting in complete or incomplete paralysis (6–8).

Therefore, reducing the leakage rate of bone cement and improving the safety of PKP surgery remain urgent clinical issues (8–11). Previous studies have systematically investigated intraoperative imaging predictors of bone cement leakage into the spinal canal, identifying risk factors such as fracture morphology, vertebral collapse severity, cortical integrity status, and preexisting fissure patterns (3, 6, 7). However, a critical gap remains in defining the safe diffusion boundary—the maximum cement distribution volume within cancellous bone that avoids extravasation—a parameter with direct implications for optimizing vertebroplasty efficacy while mitigating neurological risks. This study introduces a quantitative framework to establish the imaging-guided thresholds for cement dispersion limits, thereby advancing beyond descriptive risk analyses to provide actionable intraoperative benchmarks.

In PKP surgery, if the possibility of bone cement leakage can be predicted in advance through x-ray fluoroscopic images, the risk of intravertebral canal leakage of bone cement can be significantly reduced. In PKP, does there exist a demarcation boundary (as the “**warning line**”) where bone cement distribution within this boundary significantly reduces leakage rates, whereas cement dispersion beyond this boundary correlates with a marked increase in leakage risk? The purposes of this study are as follows: (1) To screen cases where bone cement dispersion approaches the posterior edge of the vertebrae on intraoperative x-ray fluoroscopy images and to observe postoperative spinal CT images to calculate the actual incidence of posterior wall leakage of bone cement; (2) Patients whose bone cement dispersion reached the apex of the posterior edge depression on postoperative axial CT images without leakage were selected, and the distance between the posterior edge of the bone cement dispersion and the posterior edge of the vertebral body on corresponding intraoperative x-ray lateral fluoroscopic images was measured. The aim of this study was to explore the “warning line”for posterior bone cement dispersion in intraoperative x-ray images during PKP surgery.

## Materials and methods

### Patient selection

The study subjects were selected from patients with OVCFs who underwent PKP surgery at our research institute between February 2018 and September 2022. **The inclusion criteria** for patients were as follows: (1) osteoporotic thoracic or lumbar vertebral compression fractures with a bone density T score ≤−1.0; (2) no preoperative neurological symptoms; (3) disease duration ≤3 weeks; (4) clear history of low back pain with a visual analogue scale (VAS) score ≥4; (5) surgery performed by the same surgeon via unilateral puncture PKP; and (6) intraoperative x-ray fluoroscopy lateral images showing bone cement dispersion reaching or approaching the posterior vertebral edge, as measured by two doctors. **The exclusion criteria were as follows:** (1) surgically confirmed infectious or pathological fractures; (2) congenital vertebral anomalies (e.g., hemivertebrae or congenital fusion vertebrae); (3) presence of preoperative neurological symptoms; (4) nonosteoporotic vertebral fractures; (5) inability to clearly identify the posterior vertebral wall for any reason; and (6) previous vertebral fractures or fractures with a disease duration >3 weeks. A total of 88 patients (106 vertebral bodies) meeting these criteria were included.

### Surgical operation

Patients were positioned prone, and local anaesthesia was administered at the puncture site. Under x-ray fluoroscopy, the puncture needle and cannula were carefully inserted into the vertebral body. The puncture process should avoid areas close to cortical defects, especially those in the posterior vertebral wall. Balloon expansion and high-viscosity cement injection were performed under x-ray fluoroscopy, starting approximately 1 min and 30 s after mixing. The degree of cement dispersion was closely monitored, and the injection was stopped if the cement approached or exceeded the posterior vertebral edge. Finally, the cement delivery device and cannula were removed, and the incision was compressed for hemostasis and covered with a dressing. All procedures were performed by the same surgeon via a unilateral puncture technique. Intraoperative x-ray fluoroscopy was used to ensure symmetrical pedicles in the anteroposterior view and parallel endplates in both the anteroposterior and lateral views.

### Data collection

Clinical baseline data, including age, sex, bone density, surgical segment, balloon pressure, contrast dose, and bone cement volume, were recorded during the perioperative period. The VAS score before and after surgery and postoperative neurological complications were recorded.

### Radiological measurement and indicators

All 88 patients underwent three-dimensional CT scans of the thoracic/lumbar spine after PKP surgery. The thoracic/lumbar CT Dicom data were imported into the workstation for reconstruction. All intraoperative x-ray and CT images were collected. First, patients whose bone cement dispersion approached the posterior vertebral edge were identified from intraoperative x-ray fluoroscopy images. Second, postoperative three-dimensional axial CT images were used to statistically analyse the actual degree of posterior vertebral wall leakage. Third, patients without leakage at the apex of the posterior edge dispersion were classified as Group A; those patients with leakage were classified as Group B.

On the basis of the intraoperative x-ray lateral fluoroscopy images, the posterior vertebral wall was divided into three equal parts. The patients were divided into the following groups according to the position of the bone cement dispersion in the sagittal view: Group I: dispersion edge in the upper third; Group II: dispersion edge in the middle third; and Group III: dispersion edge in the lower third. The number of patients in each group was counted, and posterior vertebral wall leakage was statistically analysed via three-dimensional CT reconstruction. The leakage rate of bone cement in each subgroup was calculated.

**Figure 1:**
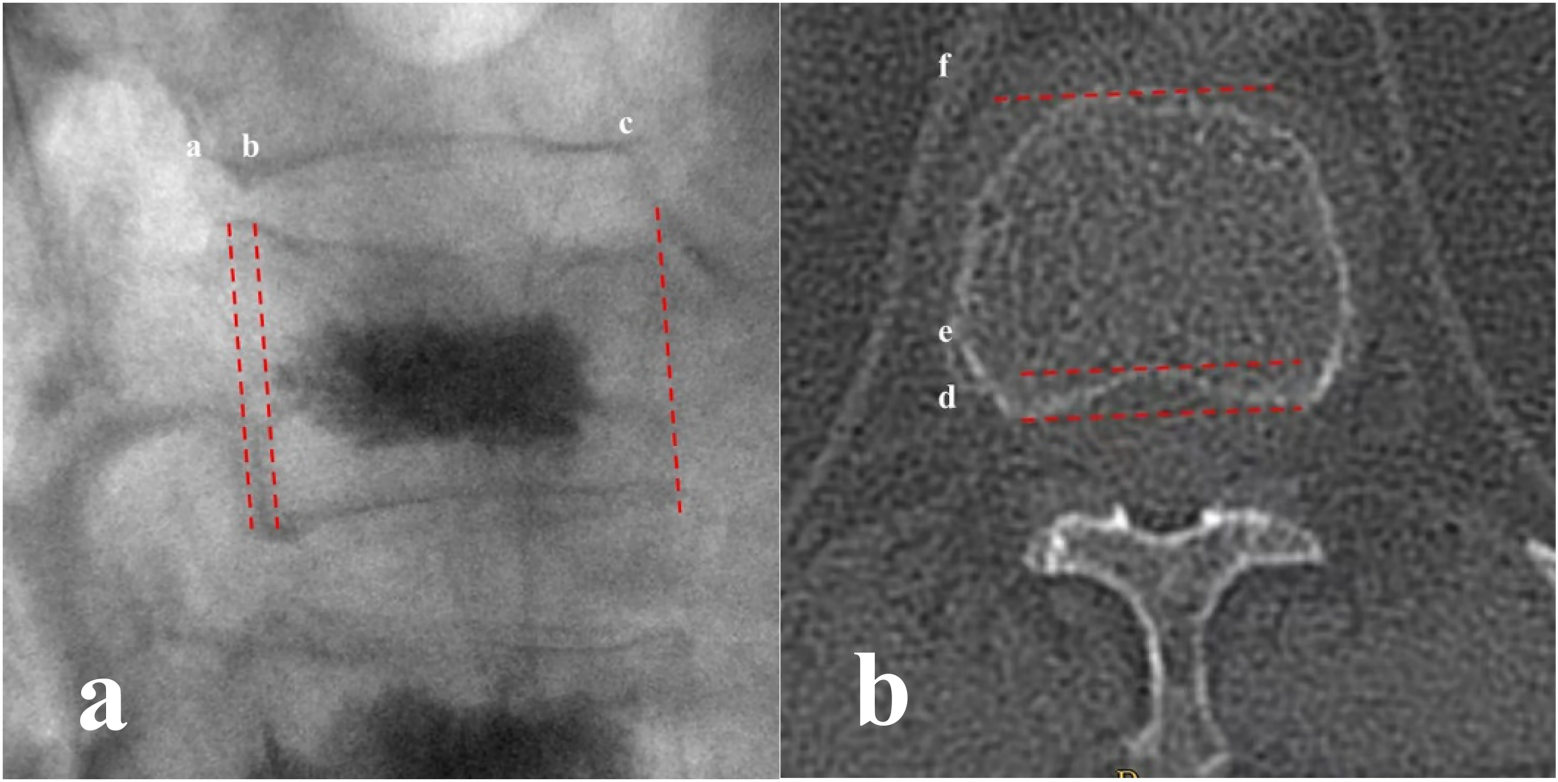
**(a)** In the intraoperative x-ray lateral fluoroscopy image, line a represents the posterior vertebral edge, line b represents a parallel line through the apex of the posterior edge of the bone cement dispersion, and line c represents the anterior vertebral edge line; **(b)** in the reconstructed CT axial images, the cortical apexes on both sides of the posterior vertebral edge were identified and connected to form line d. Lines e and f were drawn parallel to line d through the apex of the posterior vertebral depression and the anterior vertebral edge, respectively.

In the intraoperative x-ray lateral fluoroscopy image, line a represents the posterior vertebral edge, line b represents a parallel line through the apex of the posterior edge of the bone cement dispersion, and line c represents the anterior vertebral edge line. Line b is defined as “warning line” for bone cement posterior dispersion. When the bone cement reaches the apex of the posterior vertebral depression on axial CT, the warning line stays at the position on x-ray lateral fluoroscopy. The distance ab between line a and line b and the distance ac between line a and line c were measured (Figure 1). In the reconstructed axial CT images, the cortical apexes on both sides of the posterior vertebral edge were identified and connected to form line d. Lines e and f were drawn parallel to line d through the apexes of the posterior vertebral depression and the anterior vertebral edge, respectively. The distance de between Line e and Line d and the distance df between Lines f and d were measured (Figure 1). The following ratios were calculated: ab/ac for x-ray images and de/df for CT axial images. The relationships between ab/ac and de/df were compared.

### Statistical analysis

Statistical analysis was performed via SPSS 19.0 software. The measurement data were first tested for normality. If the samples conformed to a normal distribution, an independent samples t test or analysis of variance (ANOVA) was used to compare the mean values across groups to determine if there were statistically significant differences. The significance level was set at *α* = 0.05.

## Results

### Clinical outcomes

From February 2018 to September 2022, a total of 88 patients (19 males and 69 females) meeting the inclusion criteria were enrolled, encompassing 106 vertebrae. All patients underwent unilateral PKP surgery without any neurological complications postoperatively and underwent relevant preoperative and postoperative imaging examinations. The average age of the patients was 73.58 ± 9.59 years (range 52–89 years), with a bone mineral density (BMD) T score of −3.59 ± 0.86 (range −2.5 to 5.7). The distribution of fracture segments was as follows: T5 (1 case), T6 (2 cases), T7 (2 cases), T9 (4 cases), T10 (4 cases), T11 (13 cases), T12 (31 cases), L1 (17 cases), L2 (22 cases), L3 (6 cases), and L4 (4 cases). There were 73 cases of single-segment fractures and 15 cases of double- or multiple-segment fractures. The preoperative VAS score was 5.69 ± 1.15, and the postoperative VAS score was 1.20 ± 0.79 (Table 1).

**Table 1:** Clinical data of all patients.

Index	General data
Total number	88
Male	19
Female	69
Vertebra	106
Mean age	73.58 ± 9.59
Mean BMD	−3.59 ± 0.86
Fracture segment	
T5	1
T6	2
T7	2
T9	4
T10	4
T11	13
T12	31
L1	13
L2	22
L3	6
L4	4
Single-segment fracture	73
Double or multiple segment fractures	15
Preoperative VAS score	5.69 ± 1.15
Postoperative VAS score	1.20 ± 0.79
*P* (VAS)	0.00*

* Statistically significant differences, with *P* < 0.05.

### Radiological results

Postoperative 3D CT reconstruction revealed 44 patients in Group A and 62 patients in Group B, with an overall incidence of posterior vertebral wall cement leakage of 58.5%. There were no statistically significant differences between the two groups in terms of sex, age, bone density, balloon pressure, contrast agent volume, or bone cement injection volume (*P* > 0.05) (Table 2).

**Table 2:** Comparison between Group A and Group B.

Category	Age	BMD	Balloon pressure	Contrast agent volume	Bone cement volume
Group A	72.12 ± 9.51	−3.76 ± 0.79	189.03 ± 48.33	2.85 ± 0.62	3.68 ± 1.14
Group B	74.18 ± 9.61	−3.52 ± 0.88	190.00 ± 39.11	2.83 ± 0.52	3.47 ± 1.00
(F value, *P*)	(0.000, 0.317)*	(0.105, 0.187)*	(1.790, 0.914)*	(0.840, 0.811)*	(1.295, 0.331)*

* There was no statistically significant difference, with *P* > 0.05.

According to the postoperative three-dimensional CT scans, the posterior vertebral wall was divided into three equal parts. All 106 vertebral bodies were divided into three groups on the basis of the diffusion of bone cement in the posterior wall of the vertebral body: Group I (upper 1/3), Group II (middle 1/3), and Group III (lower 1/3). There were 8 vertebral bodies in Group I, with 2 vertebral bodies with posterior vertebral wall leakage, resulting in a leakage rate of 25%. Group II comprised 95 vertebral bodies, with 58 vertebral bodies with posterior vertebral wall leakage, resulting in a leakage rate of 61.1%. Group III consisted of 3 vertebral bodies, with 2 vertebral bodies with posterior vertebral wall leakage, resulting in a leakage rate of 66.7%. There were statistically significant differences in leakage rates among the three groups (*P* < 0.05). There were no statistically significant differences among the groups in terms of sex, age, bone density, balloon pressure, contrast agent volume, or bone cement injection volume (*P* > 0.05) (Table 3).

**Table 3:** Comparisons among groups I, II and III.

Classification	Age	BMD	Balloon pressure	Contrast agent volume	Bone cement volume
Group I	76.00 ± 8.90	−3.90 ± 0.93	202.50 ± 58.98	2.56 ± 0.50	3.44 ± 1.40
Group II	73.02 ± 9.57	−3.57 ± 0.86	188.00 ± 40.41	2.85 ± 0.55	3.52 ± 1.02
Group III	85.00 ± 2.65	−3.53 ± 0.91	210.00 ± 30.06	3.00 ± 0.00	4.17 ± 1.04
(F value, *P*)	(2.623, 0.077)*	(0.562, 0.572)*	(0.805, 0.450)*	(1.185, 0.310)*	(0.590, 0.566)*

* There was no statistically significant difference, with *P* > 0.05.

### Warning line in PKP surgery

**Figure 2:**
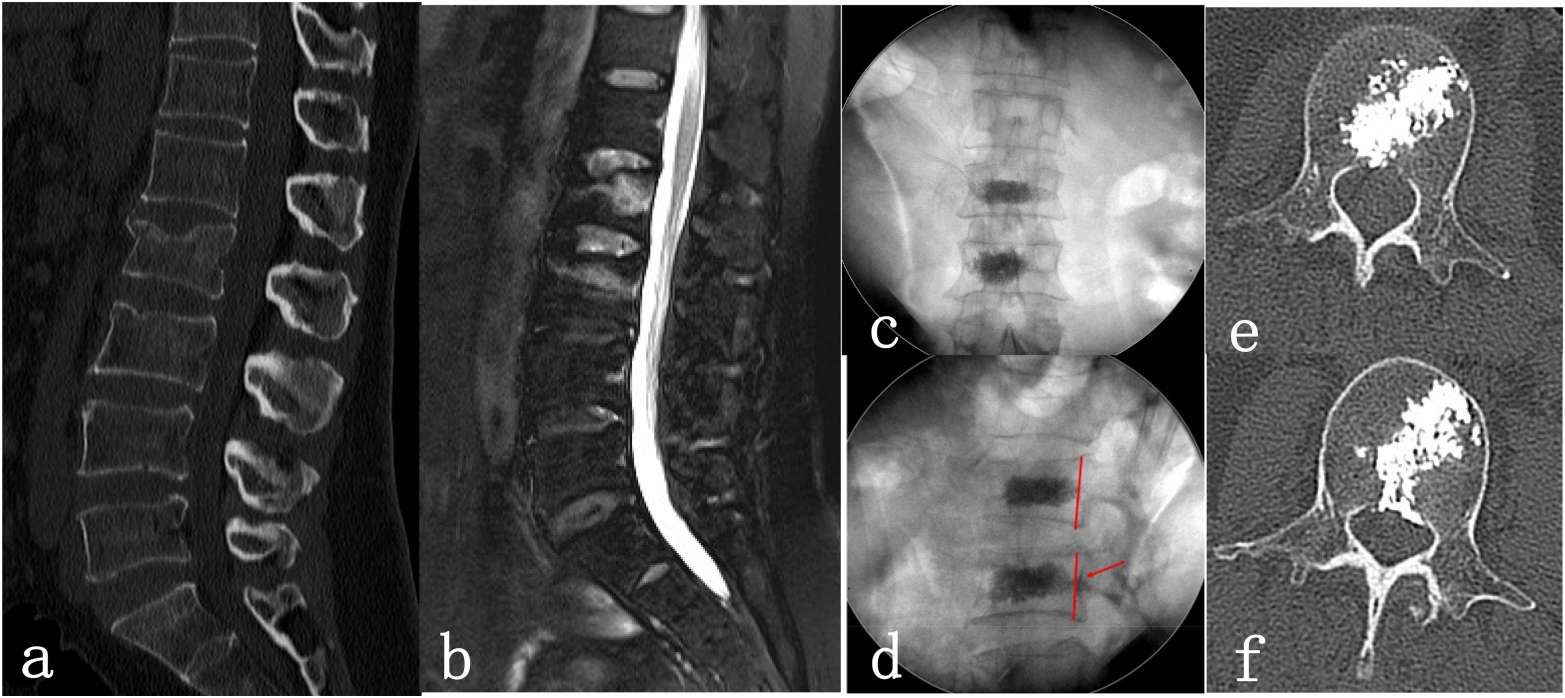
Female patient, 54 years old, suffered from low back pain and limited spinal movement after the fall injury for 2 days. She underwent unilateral percutaneous kyphoplasty (PKP) at L2 and L3. **(a,b)**: Preoperative lumbar CT showed L2 and L3 vertebral fracture lines, and preoperative lumbar MRI showed high signal in the L2 and L3 vertebral bodies; **(c,d)**: Intraoperative x-ray images, lateral fluoroscopy showed L2 bone cement diffusing backwards just enough to reach the warning line (red line), and L3 bone cement diffusing backwards beyond the warning line; **(e,f)**: Postoperative lumbar CT showed that the bone cement in the L2 vertebral body had just spread backwards and reached the posterior wall of the vertebral body without leakage, while the bone cement in the L3 vertebral body showed leakage within the spinal canal.

For the 44 vertebral bodies in group A without bone cement leakage, the ab/ac ratio was calculated to be (6.8 ± 2.17)% in the intraoperative x-ray lateral images. In the postoperative three-dimensional CT reconstructed axial image, the de/df ratio was calculated to be 11.19 ± 3.47%, which was significantly different from the ab/ac ratio (*P* < 0.05). Therefore, in PKP surgery, the location of the warning line for preventing bone cement leakage is considered to be approximately 6.8% of the sagittal diameter from the posterior edge of the vertebral body. A typical case is described in Figure 2.

## Discussion

OVCFs are a common type of fragility fracture caused by decreased bone mass. Surgical treatment is a primary approach (2, 3), aiming to quickly alleviate back pain, end prolonged bed rest, reduce the incidence of complications such as lower limb venous thrombosis, pneumonia, and pressure sores, and prevent muscle weakness or atrophy due to long-term bed rest, which can further exacerbate bone loss and osteoporosis. Among surgical treatments, PKP is widely applied for osteoporotic vertebral fractures (2, 4). Compared with traditional open surgery, PKP is advantageous because of its shorter duration, less trauma, minimal bleeding, and rapid symptom relief.

Cement leakage remains the main complication of PKP, with an incidence rate of 25% reported in the literature (12). Leakage into blood vessels or the spinal canal can lead to severe complications, including pulmonary embolism and spinal cord or nerve damage, significantly affecting surgical outcomes and reducing patients' quality of life (13–15). Therefore, to increase surgical safety, many risk factors for cement leakage have been identified, such as low bone density, hypertension, degree of vertebral compression, presence of vertebral fissures, and cement viscosity (16–18). However, there is limited research on the early detection and prevention of leakage during surgery. It is challenging to detect early leakage through intraoperative x-ray fluoroscopic lateral views (19). Wang et al. (7) noted that the anatomical feature of the posterior vertebral wall appearing arch shaped and concave inwards on axial CT images makes it difficult to detect cement leakage in a timely and accurate manner through x-ray fluoroscopy during surgery unless CT scans are performed intraoperatively. Yeom et al. (19) reported that only 7% of intrathecal cement leakage could be detected via lateral views and suggested that cement injection be stopped immediately when it reaches one-fifth of the vertebral body via intraoperative fluoroscopy. However, this indicator is subjectively defined and not an objective measurement.

In this study, cases where cement dispersion just reached the posterior edge without leakage were screened via axial postoperative spinal CT images. Measurements of CT axial images and intraoperative fluoroscopic lateral views revealed that the postoperative fluoroscopic measurement results were significantly smaller than the CT measurements were, indicating statistical significance. Zhang et al. (8) precisely measured the depth of the posterior vertebral concavity via CT. We believe that CT measurements cannot fully represent intraoperative fluoroscopic images because of differences in defining the posterior vertebral edge between the two imaging methods. Intraoperative x-ray fluoroscopic images are more intuitive and immediately useful for reference during surgery. Therefore, on the basis of our study results, we suggest that in intraoperative x-ray fluoroscopic lateral views, the warning line for cement dispersion is approximately 6.8% of the sagittal diameter from the posterior vertebral edge.

In actual cases, further observation of postoperative CT and x-ray images revealed that when the posterior vertebral wall was divided into thirds, cement leakage occurred mainly in the middle and lower thirds, with the lowest incidence in the upper third. These findings suggest that the vertebral venous foramen is a high-risk area for posterior vertebral wall cement leakage. Li et al. (20) reported that the presence of the vertebral venous foramen and relatively sparse trabeculae make the middle region of the vertebral body mechanically weakest. During vertebral compression, the central trabeculae are most severely damaged, with the largest intertrabecular distance, thus resulting in the highest leakage rate (21). In some cases, although the degree of cement dispersion exceeded the posterior vertebral edge, further observation of axial postoperative CT images revealed that the cement did not leak into the spinal canal but rather dispersed into the pedicle, possibly explaining the lower incidence of upper third leakage. Therefore, when cement dispersion exceeds the warning line in the middle and lower thirds of the vertebral body, there is a greater risk of posterior wall leakage.

PKP, as a minimally invasive treatment for OVCFs, carries the risk of intraspinal bone cement leakage that may induce severe complications like nerve compression (6). Thorough preoperative disclosure of such potential risks to patients represents not only a legal obligation but also an embodiment of ethical responsibility (22). Surgeons must specifically explain leakage probabilities and consequences based on individual bone quality, fracture anatomy, and surgical approach, avoiding generalized risk descriptions that undermine patient decision-making capacity (8, 23). Technically, scientific preventive measures should be emphasized: optimizing puncture trajectories through high-precision imaging guidance, selecting high-viscosity cement with controlled injection pressure, and employing staged filling techniques to monitor dispersion. The possibility of secondary interventions, such as emergency decompression for neurological symptoms caused by leakage, must be clarified. Authentic informed consent requires surgeons to transcend formalized signing procedures by utilizing visual models or case imaging to help patients intuitively comprehend risk gradients, thereby establishing therapeutic alliances through empathetic communication (22, 23). Only by translating technical details into perceptible decision-making references can dynamic equilibrium between medical autonomy and risk prevention be achieved.

PKP is relatively safe for treating OVCFs. The warning line for bone cement dispersion is approximately 6.8% from the posterior edge of the vertebral body. Most leaks occur in the middle and lower thirds, so caution is needed when bone cement dispersion exceeds the warning line in these areas. This study has certain limitations. First, this was a single-center retrospective study, and further validation through large-scale, multicenter research is needed. Second, the clarity of intraoperative fluoroscopic images is limited, leading to some measurement errors. Additionally, the morphology of fractured vertebrae may differ from that of normal vertebrae.

## Conclusions

PKP is a relatively safe treatment for OVCFs. In this study, the incidence of posterior vertebral wall cement leakage was 58.5%. Most bone cement leakage occurs in the middle and lower thirds of the posterior vertebral wall. Intraoperative x-ray fluoroscopic lateral measurements suggest that the warning line for cement dispersion is approximately 6.8% of the sagittal diameter from the posterior vertebral edge. When the apex of cement diffusion remains anterior to the warning line in PKP surgery, the posterior vertebral wall cement leakage rate is low. The warning line serves as a real-time fluoroscopic predictor of cement leakage risk during PKP surgery. Further analysis of the imaging results indicated that when cement dispersion exceeds the warning line in the middle and lower thirds of the vertebral body, there is a greater risk of posterior wall leakage.

## Data Availability

The original contributions presented in the study are included in the article/Supplementary Material, further inquiries can be directed to the corresponding author.
